# Ruptured Visceral Artery Aneurysms: A Deadly Cause of Epigastric Pain

**DOI:** 10.5811/cpcem.2019.1.41057

**Published:** 2019-02-26

**Authors:** Sara Bradley, Faith Quenzer, Micah Wittler

**Affiliations:** * Western University of Health Sciences, College of Osteopathic Medicine of the Pacific, Pomona, California; † Desert Regional Medical Center, Department of Emergency Medicine, Palm Springs, California

## Abstract

Visceral artery aneurysms (VAA) are rare, life-threatening disease processes that often affect the celiac, superior mesenteric, or inferior mesenteric arteries and their respective branches. The splenic, hepatic, superior mesenteric, and tripod celiac arteries are most commonly affected and have high rupture and mortality rates. This case describes splenic and celiac artery aneurysms in a patient that led to hemorrhagic shock and multisystem organ failure despite timely diagnosis and ligation. A brief review of the literature further elucidates the key risk factors in identifying patients with VAAs and their treatment course.

## INTRODUCTION

Visceral artery aneurysms (VAA) are life-threatening and often require immediate intervention upon rupture. The arteries most commonly affected by VAAs are the following: hepatic, splenic, superior mesenteric, and the celiac. Each vessel has unique risk factors and epidemiologic characteristics that can help to narrow down which of the four may be involved. Risk of rupture and mortality rates of these vessels are high, especially in pregnancy. Computed tomographic angiography (CTA) is needed in conjunction with a thorough history and physical exam to help confirm the diagnosis. Operative management is done by open surgical repair, endovascular techniques, or laparoscopic surgery.

## CASE REPORT

A 77-year-old, obese, Caucasian male presented to the emergency department (ED) with a sudden onset of lower chest and epigastric pain and sudden collapse after lifting a heavy object while working on his ranch. Per his wife, the patient was a previously healthy and active individual who had lost 50 pounds over the prior year on a diet and exercise regimen. The patient had a past medical history of gastroesophageal reflux disease, hyperlipidemia, diabetes, and hypertension. He was a former smoker from about age 15 to 60. The patient also had a history of daily alcohol use, which ended in his mid-forties.

Upon arrival to the ED, the initial vital signs revealed a blood pressure of 94/72 millimeters of mercury (mmHg), heart rate of 89 beats per minute (bpm), respiratory rate of 16 breaths per minute (BPM), and oxygen saturation of 100% on room air. On physical exam, the patient was somnolent but easily aroused, pale, and in severe distress. The cardiovascular exam revealed that the heart had regular rate and rhythm without murmurs. His lungs were clear and without wheezes, rhonchi, or rales. His abdominal exam was notable for a soft, distended, moderately tender epigastric region but without rebound or guarding. A pulsatile mass was not palpated and there were no abdominal bruits.

His initial complete blood count demonstrated a white count of 24.0 ×10^9^/L, hemoglobin of 11,000 grams per liter, platelet count of 198 ×10^9^/L, with 93% neutrophils. The comprehensive metabolic panel was unremarkable. The creatine phosphokinase and troponin were normal. Amylase and lipase were normal. An electrocardiogram (ECG) showed a sinus rhythm with no acute ST changes and a right bundle branch block, which was seen on a previous ECG.

**Image 1:**
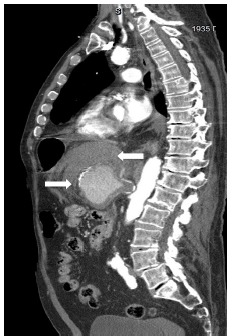
Computed tomographic angiography of the chest and abdomen demonstrating active extravasation of the celiac artery and splenic artery aneurysm (rightward arrow) with extensive stranding (leftward arrow), representing active hemorrhage.

**Image 2:**
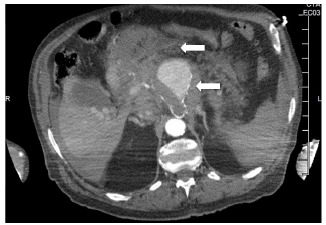
Computed tomographic angiography of the abdomen in axial view demonstrating active extravasation (top arrow) of the celiac artery aneurysm (bottom arrow).

The patient underwent a computed tomography (CT) angiogram of the chest and abdomen which showed 8.0 centimeter (cm) × 6.0 cm × 6.5 cm aneurysm in the expected location of the celiac artery and splenic artery with extensive stranding of the surrounding fat, representing active hemorrhage as seen on the sagittal abdominal CT (Images 1 and 2).

Within 30 minutes of arrival to our ED, the patient was in hypovolemic shock with hypotension (59/34 mmHg), tachycardia (142 bpm), and tachypnea (rate 26 BPM). The vascular surgeon was notified immediately and the patient was taken to the operating room within 45 minutes of arrival to undergo a ligation of the neck of the aneurysm. Upon arrival to the intensive care unit, the patient lost his pulses. He was unfortunately pronounced dead after unsuccessful heroic efforts.

## DISCUSSION

VAAs are those affecting the celiac, superior mesenteric, or inferior mesenteric arteries and their branches. These aneurysms are uncommon compared to aortic or iliac. Splenic (60%), hepatic (20%), superior mesenteric (5.9%) and tripod celiac (4%) are the most common arteries affected.^1^ Visceral aneurysms have a low incidence rate between 0.1% and 0.2%, and a high rupture rate (25%) compared to aortic and iliac. Twenty-two percent of these aneurysms present as clinical emergencies, with a 70% mortality rate.^2^ This is a rare case in which our patient had an aneurysm affecting both the celiac and splenic arteries. Incidence rates for celiac aneurysms are approximately 0.01%. There is no gender predilection, but some studies have indicated more frequent occurrence in males compared to females. Celiac aneurysms are commonly diagnosed in the fifth decade of life.^3^

**CPC-EM Capsule**

What do we already know about this clinical entity?

*Visceral artery aneurysms (VAA) are rare and often affect the celiac, superior mesenteric, or inferior mesenteric arteries and their respective branches*.

What makes this presentation of disease reportable?

*Diagnosis of VAAs can be elusive due to the subtle presentation of symptoms that can mimic other diseases. However, when ruptured, they can be catastrophic*.

What is the major learning point?

*Ruptured VAA should be considered in the differential diagnosis in a patient with cardiovascular collapse and epigastric pain*.

How might this improve emergency medicine practice?

*An awareness and understanding of VAAs may potentially improve morbidity and mortality with early detection of the disease*.

These aneurysms are usually symptomatic at the time of diagnosis but can be asymptomatic as well, and are commonly found in association with other aneurysms, such as aortic, renal, popliteal, and femoral. The most common presenting symptoms are epigastric pain, back pain, nausea, abdominal distention, hematochezia, hematemesis, or a palpable pulsatile mass.^4^ The presentation can commonly mimic pancreatitis given the location of the vessel. The exact etiology of celiac aneurysms is unknown, but they are frequently associated with syphilis, tuberculosis, arteriosclerosis, medial degeneration, fibrous dysplasia, trauma, or mycotic lesions. Approximately 42% are idiopathic in nature.^5^

The risk of rupture for celiac aneurysms is about 13%.^6^ Ruptured celiac artery aneurysms have a mortality rate of approximately 80%, while the mortality rate of non-ruptured aneurysms ranges from 5–10%. Risk factors for rupture include pregnancy and increased diameter (>20 millimeters [mm]).^4^,^5^ Early diagnosis is crucial, as the emergency operative mortality rate is 40% vs elective (5%).^5^ Criteria for intervention in asymptomatic patients include the following: aneurysms greater than 2 cm in diameter with sensible operative risk; radiologic evidence of increasing aneurysm size; or a size greater than 3–4 times the original diameter of the vessel.^6^

Splenic artery aneurysms (SAA) are the most common true abdominal aneurysm behind aortic and iliac and are found four times more often in females than in males, occurring most commonly in patients in their fifth or sixth decade of life.^7^ Autopsy studies indicate an incidence of SAA between 0.1% and 10.4% and association with intra-abdominal aneurysms involving other visceral arteries. The reported rate of rupture is between 3% and 9.6%, with a mortality of 36% after rupture.^8^ The etiology of SAAs is unknown, but are frequently seen in association with portal hypertension, pregnancy, multiparity, arterial venous fistulas and malformations, atherosclerosis, hypertension, liver transplantation, and cirrhosis. Occurrence of SAA can be seen in 7%–17% of chronic liver disease patients with cirrhosis.^9^ Eighty percent of these aneurysms are found to have atherosclerotic changes and calcification.^10^ Incidence of rupture is seen most frequently in young, pregnant women, with an associated mortality of 75% and a fetal mortality of 95%. Patients with unruptured SAAs are commonly asymptomatic, and are diagnosed incidentally. The most common clinical complaint is epigastric abdominal pain, but patients can also present with splenomegaly, palpable pulsatile mass, hematochezia, melena, chest pain, or nausea.

Hepatic artery aneurysms (HAA) are the most commonly reported visceral pseudoaneurysm and have a mortality rate of 40%. They have an incidence rate of 0.02–4.0%, as well as a rupture rate of 80%.^11^ HAAs are associated with atherosclerosis, cystic medial necrosis, trauma, mycotic embolization, trauma, Marfan syndrome, Klippel-Trenaunay syndrome, or giant cell arteritis.^12^ Patients with aneurysms from nonatherosclerotic etiologies are at higher risk of rupture. Fifty-five percent of these patients present with abdominal pain, and gastrointestinal hemorrhage occurs in up to 46%.^13^ Hemobilia, which is described by Quincke’s triad (jaundice, biliary colic, and gastrointestinal bleeding) occurs in one-third of patients with HAA.^11^ Surgical intervention is recommended when the aneurysm reaches greater than 20.0 mm.^12^ Patients with superior mesenteric artery (SMA) aneurysms are usually symptomatic and can present with intermittent abdominal pain prior to rupture. These aneurysms commonly have mural thrombosis, which may ultimately result in acute mesenteric ischemia. SMAs can also invade into adjacent visceral organs, resulting in severe hemorrhage.^13^ SMAs are associated with higher dissection rates than the other visceral artery aneurysms, but only have a reported incidence of 0.06%.^14^ The rate of rupture for SMA aneurysms is 50%, where initial symptoms include hypovolemic shock, hemoperitoneum, or acute abdominal pain. These lesions are associated with high emergency surgery mortality rates (20–40%).^15^ Risk factors include arteriosclerosis, septic emboli, mycotic disease, pancreatitis, connective tissue disease, and trauma.^16^ It is important to quickly diagnose and treat these aneurysms to prevent serious complications including gastrointestinal or intraperitoneal hemorrhage, thrombosis, distal embolization, arterial spasm, arteriovenous fistula formation, secondary portal hypertension, bowel infarction, and death.^15^

An emergency physician’s main role in the care of a patient with an acutely ruptured VAA is swift diagnosis and immediate surgical consultation. Prior to surgical intervention, standard resuscitative measures (insertion of two, large-bore intravenous catheters, cardiac monitoring, and supplemental oxygen) as well as preparation for administration of blood products should be done. Fluid resuscitation is needed if the patient is hemodynamically unstable; however, over-resuscitation has the potential to worsen active bleeding. Imaging modalities for diagnosis of VAA include ultrasound, CT with angiography, magnetic resonance imaging, and abdominal aortic arteriography being the gold standard. Point-of-care ultrasound can be useful to quickly visualize the aneurysm and associated free fluid inside the abdomen, especially for patients who are unstable and cannot undergo CT. 17

Surgical intervention for VAAs varies and depends on the clinical presentation, etiology, the patient’s comorbidities, and location of the aneurysm. The various techniques include open surgical repair, endovascular treatment, and laparoscopic surgery.^11^ Indications for treatment of VAA include presence of pseudoaneurysm, symptomatic VAA, asymptomatic VAA greater than 2 cm, rapid expansion (greater than 0.5 cm per year), pregnancy, women of childbearing age, or liver transplantation.^3^ Notably, the threshold for intervention is significantly lower than that of aneurysms involving the aorta (greater than 5.5 cm). The summary table synthesizes the epidemiology associated with each type of VAA along with presenting symptoms, morbidity, and mortality (Table).

## CONCLUSION

Ruptured VAA is not often considered in the differential diagnosis of abdominal pain or, more importantly, in sudden collapse. When approaching a patient with signs of abdominal vascular catastrophe, differential diagnosis should include visceral as well as aortic aneurysms. VAAs are often not suspected initially in patients presenting with abdominal complaints, given their low prevalence, and this can delay diagnosis. However, in a patient with hypotension and epigastric abdominal pain, a life-threatening hemorrhage secondary to VAA should be suspected. One should be especially concerned for rupture in the pregnant or postpartum population. The patient in this case report had risk factors for VAA, including age, gender, past smoking and alcohol use, which may have predisposed him to atherosclerosis. Due to the high rate of rupture, the typical approach to VAA is early elective intervention, but in profound hypotension and suspected rupture, the patient will likely need emergent vascular surgery.

## Figures and Tables

**Table:** Visceral artery aneurysms.

Vessel	Epidemiology	Risk factors	Presentation	Rate and risk for rupture	Mortality rate
Celiac artery	Males, 5th decade of life	Syphilis Tuberculosis Arteriosclerosis	Epigastric abdominal pain Back pain Palpable pulsatile mass	Rate of rupture: 13%	80%
	Incidence: 0.01%	Medial degeneration Fibrous dysplasia Trauma Mycotic lesions	Hematochezia/Melena Chest pain Nausea Mimics pancreatitis	Risk increases with pregnancy and diameter >20mm	
Splenic artery	Females, 5th or 6th decade of life	Portal hypertension Pregnancy/multiparity Atherosclerosis	Epigastric abdominal pain Splenomegaly Palpable pulsatile mass	Rate of rupture: 3–9.6%	36%
	Incidence: 0.1% – 10.4%	Arterial venous fistulas and malformation Hypertension Liver transplantation/cirrhosis	Hematochezia/Melena Chest pain Nausea	Most commonly ruptures in young pregnant women	
Hepatic artery	Males	Arteriosclerosis Cystic medial necrosis	Abdominal pain Hematemesis	Rate of rupture: 80%	40%
	Incidence: 0.02–4.0%	Trauma Mycotic embolization Marfan syndrome Klippel-Trenaunay syndrome Giant cell arteritis	Jaundice Portal hypertension		
Superior mesenteric artery	Males	Arteriosclerosis Septic emboli	Symptomatic prior to rupture - intermittent abdominal pain or acute mesenteric ischemia from thrombosis	Rate of rupture: 50%	20–40%
	Incidence: 0.06%	Mycotic disease Pancreatitis Connective tissue disease Trauma		High emergency surgery	
			Once ruptured, hypovolemic shock, hemoperitoneum, and acute abdominal pain		

*Mm*, millimeters.
